# CT Angiography and Presentation NIH stroke Scale in Predicting TIA in Patients Presenting with Acute Stroke Symptoms

**DOI:** 10.4172/2329-6895.1000140

**Published:** 2013-11-08

**Authors:** Bedriye Karaman, James Selph, Joselyn Burdine, Cole Blease Graham, Souvik Sen

**Affiliations:** 1 Ege University Medical School, Department of Neurology, Izmir, Turkey; 2 University of South Carolina School of Medicine, Department of Neurology Columbia, South Carolina, USA

**Keywords:** Ischemic stroke, Neuroimaging, TIA, Discharge, Clinical outcome

## Abstract

Patient candidacy for acute stroke intervention, is currently assessed using brain computed tomography angiography (CTA) evidence of significant stenosis/occlusion (SSO) with a high National Institutes of Health Stroke Scale (NIHSS) (>6). This study examined the association between CTA without significant stenosis/occlusion (NSSO) and lower NIHSS (≤ 6) with transient ischemic attack (TIA) and other good clinical outcomes at discharge. Patients presenting <8 hours from stroke symptom onset, had an NIHSS assessment and brain CTA performed at presentation. Good clinical outcomes were defined as: discharge diagnosis of TIA, modified Rankin Score [mRS] ≤ 1, and home as the discharge disposition. Eighty-five patients received both an NIHSS at presentation and a CTA at 4.2 ± 2.2 hours from stroke symptom onset. Patients with NSSO on CTA as well as those with NIHSS≤6 had better outcomes at discharge (p<0.001). NIHSS ≤ 6 were more likely than NSSO (p=0.01) to have a discharge diagnosis of TIA (p<0.001). NSSO on CTA and NIHSS ≤ 6 also correlated with fewer deaths (p<0.001). Multivariable analyses showed NSSO on CTA (Adjusted OR: 5.8 95% CI: 1.2-27.0, p=0.03) independently predicted the discharge diagnosis of TIA. Addition of NIHSS ≤ 6 to NSSO on CTA proved to be a stronger independent predictor of TIA (Adjusted OR 18.7 95% CI: 3.5-98.9, p=0.001).

## Introduction

The annual incidence of stroke in the United States is approximately 795,000 of which 610,000 are first strokes [1]. Stroke is the fourth leading cause of mortality in the United States and is the leading cause of long-term adult disability [2]. Predicting stroke outcome is important for both the patient and the healthcare providers for treatment and discharge management. Discharge clinical outcome is shown as an effective predictor of long-term outcome after stroke [3]. NIHSS has also been used in most studies to predict functional outcome after stroke [4]. Previous researchers have shown that NIHSS has a significant correlation with infarct volume [5], discharge disposition [6], and outcome after stroke [6,7].

The annual incidence of TIAs in the United States is estimated to be approximately 240,000 [8]. TIAs have been found to be a strong predictor of subsequent stroke and death [9]. Prognostic clinical scores (ABCD2 and ABCD3-I), as well as specific clinical signs and symptoms (e.g. fluctuations) have been used to predict early stroke risk in patients admitted to hospital after TIA [10]. However, these scores are of limited value in predicting if a patient with acute stroke symptoms will turn out to be a TIA with complete resolution of symptoms or a stroke with persistent symptoms and disability.

Imaging of the cerebrovascular system is being utilized to assess candidacy for acute stroke intervention. CTA provides a quick non-invasive assessment of the intracranial and extracranial circulation [11]. Examining the intracranial vasculature of acute stroke symptoms may assist in the treatment decision [12]. Proximal occlusion shown on CTA has been correlated with poor response to systemic IV-tPA and poor outcome after ischemic stroke [13]. Prior studies have investigated a combination of NIHSS and CTA findings to predict poor outcomes [14,15]. NIHSS is associated with excellent inter-rater reliability (Kappa=0.95) [16] whereas CTA is associated with a moderately good inter-rater reliability (Kappa=0.63) [17]. The purpose of this study was to evaluate the ability of CTA with and without NIHSS to independently predict TIA, and to evaluate whether CTA and NIHSS correlate with good discharge outcomes.

## Methods

Approval for this research was first obtained from the local Institutional Review Board. A retrospective medical chart review was conducted for patients presenting at Palmetto Health Richland Hospital with acute ischemic stroke symptoms in a 24-month period (January 2010-December 2011). Included were patients with acute ischemic stroke/TIA with CTA performed within eight hours from symptom onset. The following items were collected on patients: known stroke prognostic factors, NIHSS on presentation, status of IV-tPA administration prior to obtaining CTA, CTA data performed as a standard of care, and discharge disposition.

The stroke prognostic factors assessed were those that have been previously shown to be associated with stroke outcome: age, male gender, non-Caucasian race, hypertension, diabetes, coronary artery disease (CAD), congestive heart failure (CHF), hyperglycemia, decreased level of consciousness on presentation, prior stroke, and stroke subtypes [16]. The initial NIHSS was performed by a certified neurologist as a part of the initial acute stroke assessment and was used to gauge stroke severity on presentation and as a prognostic marker of stroke outcome [4].

Stroke subtype was classified according to TOAST (Trial of Org 10172 in Acute Stroke Treatment) classification [17]. Patients with large artery atherothrombosis and other stroke subtypes were analyzed separately as subgroups. Significant stenosis was defined as a narrowing of ≥ 50% in a major intracranial cerebral artery. Previous studies have shown that CTA has high sensitivity and specificity for examining ≥ 50% stenosis of large intracranial arterial arteries [18]. Good outcome was defined as: a modified Rankin Scale (mRS) ≤ 1 at discharge, patients discharged home without nursing care, and a discharge diagnosis of TIA (implying complete neurological recovery at time of discharge). These discharge data were obtained from the inpatient hospitalization chart and adjudicated by a vascular neurologist.

Both CT and CTA were performed on a dual source Siemens SOMATOM Definition 64-slice CT scanner (Siemens Medical Solutions, Forchheim, Germany). Head CT scans were performed using 4.8 mm contiguous slices in a plane 20° negative to the canthomeatal plane. In the absence of hemorrhage or other contraindications for thrombolysis or contraindication for CTA (renal failure, allergy to radiocontrast agent or lack of IV access), the patients underwent a CTA. For all patients an 18G intravenous catheter was placed in vein in an arm in the emergency room as a part of the hospital's acute stroke protocol. Immediately following the head CT scan, a CTA was performed. A 60 ml bolus of nonionic radiographic contrast agent (Omnipaque 350; Nycomed Inc. /Nycomed A.S., Oslo, Norway) was given intravenously was by a power injector (Medrad, Indianola, PA, USA) at 5.5 ml/sec and 300 psi. Scanning was timed to acquire 0.6 mm axial images from the level of the aortic arch to the convexity of brain in a helical fashion (pitch .85). Axial 1 mm reconstructed images (at 0.5 mm increments) were generated. In addition, maximum intensity projection reconstructed images of the extracranial cervical and intracranial vasculature in the axial, coronal and sagital overlapping (6 mm at 2 mm increments) planes were created in approximately five minutes per plane. The images were reviewed by the neuroradiologist and/or interventionalist for evidence of extracranial or intracranial large vessel occlusion or flow limiting lesion.

Univariate analyses were performed to test inter-group differences of continuous variables. For normally distributed variables t-test was used. Categorical variables were analyzed using Fisher's exact test. These analyses examined the correlation between CTA results and good clinical outcome. Statistical analysis was performed using IBM SPSS version 20.0 (Chicago, IL). Forward conditional multiple logistic regression analyses were performed to compare brain CTA with and without NIHSS in predicting discharge diagnosis of TIA. In all analyses, p ≤ 0.05 was considered statistically significant.

## Results

A total of 85 patients were included in this study: 36 (42%) women and 49 (58%) men with a mean age of 65.8 (range 28-92 years). Of these patients, 51 (60%) were white and 34 (40%) were African American. Median NIHSS was 4.5 (IQR: 8) in all patients. Brain CTA was performed in 85 patients of whom 59 (69%) patients had stroke and 26 (31%) had TIA. Characteristics of patients with or without SSO on CTA are shown according to CTA results in Table 1 and according to NIHSS in Table 2.

Compared to patients with significant stenosis/occlusion (SSO), patients without significant stenosis/occlusion (NSSO) were less likely to have fever (p=0.004) and decreased consciousness (p=0.001) on presentation (Table 1). There were no significant differences in mean age, proportion of males, non-Caucasians, those with hypertension, diabetes, atrial fibrillation, CAD, CHF, hyperglycemia, and prior stroke. The proportion of patients receiving IV-tPA was higher in the SSO group compared to the NSSO group (p=0.003). It should be noted that per the hospital's acute stroke protocol, CTA is only obtained in selected IV-tPA patients that do not show significant clinical recovery after the tPA administration (ΔNIHSS<4). When examining etiological stroke subtypes, patients with NSSO were less likely to have large artery atherothrombosis (p=0.002) and more likely to have small vessel occlusion (p=0.002). Patients with NSSO on CTA had good outcome at discharge according to mRS (p<0.001) and disposition to home (p<0.001) at discharge. There was a significant difference in the number of patients who died with SSO (N=8) compared to those with NSSO who (N=2) died (p<0.001). The proportion of TIAs in the NSSO group (38%) was higher than those with SSO (9%), although the difference did not reach statistical significance (p=0.1).

Compared to patients with NIHSS>6, patients with NIHSS≤6 were also less likely to have fever (p=0.002) and decreased consciousness (p<0.001) on presentation (Table 1). There were no significant differences in mean age, proportion of males, non-Caucasians, those with hypertension, diabetes, atrial fibrillation, CAD, CHF, hyperglycemia and prior stroke. The proportion of patients receiving IV-tPA was higher in the NIHSS>6 group compared to the NIHSS ≤ 6 group (p<0.001). When examining etiological stroke subtypes, patients with NIHSS ≤ 6 were also less likely to have large artery atherothrombosis (p=0.02). Patients with NIHSS ≤ 6 had good outcome at discharge according to mRS (p<0.001), disposition to home (p<0.001) and TIA diagnosis (p<0.001) at discharge. There was a significant difference in the number of patients who died with NIHSS>6 (N=10) compared to those with NIHSS ≤ 6 (N=0) (p<0.001).

In univariate analyses (Table 3), NSSO on CTA was a significant predictor of TIA (p=0.001, OR: 10.5, 95% CI: 2.5-44.8). Addition of NIHSS ≤ 6 to NSSO on CTA yielded a stronger prediction of TIA (p<0.001, OR: 18.5, 95% CI: 4.5-76.8). In forward conditional logistic regression, independent predictors of TIA were NSSO on CTA, absence of hyperglycemia and lack of prior history of stroke (in that order, p<0.05 for all). NSSO on CTA was an independent predictor of TIA (p=0.03 Adjusted OR 5.8 95% CI: 1.2-27.0). On adding NIHSS ≤ 6 to NSSO on CTA and using the forward conditional logistic regression, independent predictors of TIA were NSSO on CTA, absence of hyperglycemia and lack of prior history of stroke (in that order, p<0.01 for all). Addition of NIHSS ≤ 6 to NSSO on CTA proved to be a stronger independent predictor of TIA (p= 0.001 Adjusted OR 18.7 95% CI: 3.5-98.9).

## Discussion

Several previous studies have investigated the use of CTA imaging in predicting stroke outcome. Verro et al., reported that occlusion or high-grade stenosis on brain CTA in 56% of patients presenting with acute stroke symptoms with a strong correlation with poor outcome [19]. Verro et al. published that a combination of clinical and CTA findings were a better predictor of stroke outcome than NIHSS alone [20]. Gonzalez et al. found CTA evidence of occlusion in 31% of patients with acute stroke symptoms and in combination with parenchymal ischemic changes on non-contrast CT and NIHSS predicted poor outcome [21]. None of these previous studies investigated whether CTA with or without NIHSS predicted TIA and good outcome. We found SSO on CTA in 26% of patients presenting with acute stroke symptoms and a lack of SSO (NSSO) predicted TIA and good stroke outcomes. The lower rate of abnormal CTA may be explained by the fact that these were consecutive patients with acute stroke symptoms, several of whom turned out to be TIA. The Calgary CTA group has shown that intra-extracranial stenosis or occlusion was associated with poor outcome at discharge in patients with TIA [22]. A recent study by the same group reported that early evaluation of brain and neck CTA predicted functional outcome and recurrence of vascular events after suffering a TIA [23]. These studies did not factor in other clinical indicators known to influence stroke outcomes. In this study we specifically investigated and found that brain CTA findings independently predicted TIA and were associated with good outcome at discharge. Supplementing brain CTA findings with NIHSS scores improved the independent prediction of TIA and the association with good outcome at discharge.

In patients with acute stroke symptoms, clinicians are unable to differentiate stroke from TIA based on CT scan of the head since infarcts do not appear on CT during the first 24 hours. CTA has been used clinically to assess for significant stenosis/occlusion, candidacy for stroke intervention, and has recently been shown to help prognosticate stroke and TIA patients. This study indicated that NSSO on brain CTA in patients with acute stroke symptoms were more frequently diagnosed as a TIA rather than stroke (p=0.01). NIHSS ≤ 6, on the other hand was more significantly associated with TIA diagnosis (p<0.001). In forward conditional logistic regression, NSSO on CTA was independent predictor of TIA (p=0.03 Adjusted OR 5.8 95% CI: 1.2-27.0). On adding NIHSS ≤ 6 to NSSO on CTA and using the forward conditional logistic regression, NIHSS ≤ 6 added to NSSO on CTA proved to be a stronger independent predictor of TIA (p=0.001 Adjusted OR 18.7 95% CI: 3.5-98.9).

Both Brain CTA and NIHSS ≤ 6 may help physicians treating patients with acute stroke symptoms predict discharge outcome including mRS≤1, discharge to home and death. Prior studies have determined that stroke etiological subtypes may be an important determinant of outcome and prognosis [24]. In this study, stroke subtypes other than large artery atherothrombosis were interpreted as a predictor of good outcome, while good clinical outcomes were significantly less in patients with large artery atherothrombosis. When cardiovascular risk factors were evaluated, a significant difference was not found between good and poor outcomes, although previous studies have shown that factors such as diabetes [25] and atrial fibrillation [26] were correlated with poor outcome after ischemic stroke. This lack of significant correlation may be due to a modest sample size. Consistent with prior studies we found that fever at presentation [27] and decreased level of consciousness [28], correlated with poor stroke outcome. After adjustment for these factors, NSSO on Brain CTA and NIHSS ≤ 6 maintained a significant association with good outcome at discharge.

The study had some limitations. This was a retrospective study utilizing electronic medical records. This limited the CTA examination to a single observation. The study also had a limited sample size. Subjects included in this study did not have post-discharge follow-up available for analyses. Therefore, long-term outcome and recurrent event may not be reflected by the results. In the future, a larger study which examines long-term outcome will need to be performed to verify and expand upon the results from this study.

Our results confirms the fact that NSSO on brain CTA may be associated with good outcome at discharge. Based on the results of this study, the treatment approach to these patients may be modified in order to improve clinical outcome at discharge. We focused on the importance of NIHSS at admission. Addition of neurologic examination with NIHSS to imaging modalities may provide a better means of predicting TIA, and good outcome in patients with acute ischemic stroke symptoms. If this finding is validated in a larger study, CTA along with NIHSS may have an additional value to clinicians deciding if the acute stroke patient will turn out to be a TIA requiring a quick work-up and aggressive stroke preventive strategy or a stroke requiring rehabilitation and discharge planning.

## Conclusion

NSSO on brain CTA with and without NIHSS ≤ 6 are independent predictors of TIA. NSSO on brain CTA with and without NIHSS ≤ 6 are individually associated with good outcome at discharge.

## Figures and Tables

**Table 1:** Characteristics of patients with or without SSO on brain CTA.

Study Population N=85	NSSO N=63	SSO N=22	p*
**Store Outcome Determinants**			
**Age (Mean ± SD)**	65.0 ± 15.0	70.0 ± 14.6	0.2
**Male Gender**	39 (62%)	10 (46%)	0.2
**Non-Caucasian**	26 (41%)	8 (36%)	0.8
**Hypertension**	53 (84%)	20 (91%)	0.7
**Diabetes**	23 (37%)	7 (32%)	0.8
**Atrial Fibrillation**	23 (37%)	12 (55%)	0.2
**CAD**	25 (40%)	9 (41%)	>0.9
**CHF**	21 (33%)	9 (41%)	0.6
**Hyperglycemia**	24 (38%)	12 (55%)	0.2
**Fever**	7 (11%)	9 (41%)	0.004
**Decreased consciousness**	6 (10%)	10 (46%)	0.001
**Prior stroke**	16 (25%)	2 (9%)	0.1
**IV-tPA administration**	6 (10%)	9 (41%)	0.003
**Stroke (TOAST) Subtypes**	N=39	N=20	
**Cardioembolic**	13 (33%)	6 (30%)	>0.9
**Large Artery Atherothrombosis**	3 (8%)	9 (45%)	0.002
**Small Vessel Occlusion**	20 (51%)	2 (10%)	0.002
**Others^†^**	3 (8%)	3 (15%)	0.4
**Discharge Outcome**			
**TIA**	24 (38%)	2 (9%)	0.01
**Discharge mRS ≤ 1**	43 (68%)	4 (18%)	<0.001
**Discharge Home**	39 (62%)	4 (18%)	<0.001
**Death**	2 (3%)	8 (36%)	<0.001

* Fisher's exact test; except for age (t-test),

Others: Known, unknown, and ≥ 1 cause identified categories combined for analysis due to small numbers in the subgroup.

**Table 2:** Characteristics of patients with or without NIHSS>6.

Study Population N=85	NIHSS≤6 N=52	NIHSS>6 N=33	p*
**Store Outcome Determinants**			
**Age (Mean ± SD)**	64.8 ± 14.2	68.3 ± 16.1	0.3
**Male Gender**	30 (58%)	19 (58%)	>0.9
**Non-Caucasian**	23 (44%)	11 (33%)	0.4
**Hypertension**	43 (83%)	30 (90%)	0.4
**Diabetes**	16 (31%)	14 (42%)	0.4
**Atrial Fibrillation**	19 (37%)	16 (49%)	0.4
**CAD**	20 (39%)	14 (42%)	0.8
**CHF**	17 (33%)	13 (39%)	0.6
**Hyperglycemia**	18 (35%)	18 (55%)	0.1
**Fever**	4 (8%)	12 (36%)	0.002
**Decreased consciousness**	2 (4%)	14 (42%)	<0.001
**Prior stroke**	13 (25%)	5 (15%)	0.4
**IV-tPA administration**	1 (2%)	14 (42%)	<0.001
**Stroke (TOAST) Subtypes**	N=26	N=33	
**Cardioembolic**	11 (33%)	8 (31%)	0.2
**Large Artery Atherothrombosis**	9 (27%)	3 (12%)	0.02
**Small Vessel Occlusion**	8 (24%)	4 (54%)	0.4
**Others**†	5 (15%)	1 (4%)	0.1
**Discharge Outcome**			
**TIA**	26 (50%)	0	<0.001
**Discharge mRS ≤ 1**	44 (85%)	3 (9%)	<0.001
**Discharge Home**	40 (77%)	3 (9%)	<0.001
**Death**	0	10 (30%)	<0.001

* Fisher's exact test; except for age (t-test),

† Others: Known, unknown, and ≥ 1 cause identified categories combined for analysis due to small numbers in the subgroup.

**Table 3:** Odds ratio (95% Confidence Interval) of NSSO on CTA and NIHSS ≤ 6 predicting TIA.

Type of analysis	Univariate	Multivariable*
NSSO	10.5 (2.5-44.8)	5.8 (1.2-27.0)†
NSSO+ NIHSS ≤ 6	18.5 (4.5-76.8)	18.7 (3.5-98.9)

* Covariates included were absence of factors associated with poor stroke outcome (age, gender, race, hypertension, diabetes, atrial fibrillation, CAD, CHF, hyperglycemia, fever, decreased consciousness, and prior stroke) were entered in a forward conditional multiple logistic regression model.

† Variables included in the final equation included NSSO, lack of prior stroke and absence of hyperglycemia.

‡ Variables included in the final equation included NSSO+NIHSS ≤ 6, lack of prior stroke and absence of hyperglycemia.
